# Supplementary material for: Identification of potential inflammation markers for outgrowth of cow’s milk allergy
Source: PLoS One. 2025 Sep 10;20(9):e0331462. doi: 10.1371/journal.pone.0331462 (PMC12422486; doi:10.1371/journal.pone.0331462)
Supplement: S1 File — (DOCX) [file pone.0331462.s001.docx]

**Supplementary Material for: Identification of potential inflammation markers for outgrowth of cow’s milk allergy**

Diana M. Hendrickx^1,*^, Mengyichen Long^1^, PRESTO study team^^^, Harm Wopereis^2^, Renate G. van der Molen^3,¶^, Clara Belzer^1,¶,*^

1 Laboratory of Microbiology, Wageningen University, Wageningen, The Netherlands

2 Danone Nutricia Research, Utrecht, The Netherlands

3 Department of Laboratory Medicine, Laboratory of Medical Immunology, Radboudumc, Nijmegen, The Netherlands

* Corresponding author

Email: [diana.hendrickx@wur.nl](mailto:diana.hendrickx@wur.nl) (DMH); [clara.belzer@wur.nl](mailto:clara.belzer@wur.nl) (CB)

¶ These authors contributed equally to this work.

^Membership of the PRESTO study team is provided after the Acknowledgments.

**SUPPLEMENTARY METHODS & RESULTS**

**1. Demonstration of IgE-mediated CMA**

In short, infants sensitized to CM, confirmed by CM-specific serum IgE >0.1 kU/L and/or CM skin prick test wheal size ≥3 mm, were diagnosed with CMA as follows. For some infants, CMA diagnosis was confirmed by an open or double-blind placebo-controlled CM challenge (DBPCFC). For others, diagnosis was confirmed by a history of anaphylaxis reaction to CM reported by two physicians. The criterion of CM-specific serum IgE >0.1 kU/L was always applied in combination with an open food challenge or a DBPCFC, unless the intake of cow's milk resulted in an anaphylactic reaction. The combination was necessary for eligibility.

**2. Demonstration of resolution of CMA**

Infants received a DBPCFC with CM powder, and in case this was negative an oral fresh milk challenge was performed. Infants negative for both tests were considered to have outgrown their CMA.

**3. The proximity extension assay (PEA) protocol**

In PEA, matched antibody pairs are labelled with DNA oligonucleotides. These antibody pairs bind their target proteins in the samples, which brings the DNA nucleotides in proximity to each other. The DNA nucleotides which are in proximity hybridize, and are extended by addition of a DNA polymerase. This results in the creation of DNA barcodes, which are subsequently amplified and quantified by qPCR.

**4. NPX values and between-plate normalization**

NPX is an arbitrary unit on log2 scale used by Olink®. Between-plate normalization was performed using intensity normalization v.2, which applies the median of the samples as normalization factor. More detail on this normalization method is provided on the Olink® website (<https://7074596.fs1.hubspotusercontent-na1.net/hubfs/7074596/05-white%20paper%20for%20website/1096-olink-data-normalization-white-paper.pdf> ).

**5. Quality control**

Quality control (QC) was performed by adding four internal controls to each sample. Individual samples pass the QC when their deviation from the median value of the controls < 0.3 NPX from the median.

**6. Results of the quality control and filtering**

Four samples (two from visit 6 months, two from 12 months) did not pass the QC. As these samples were not detected as outliers by principal component analysis (S1 Fig), they were not removed from the data set. Thirty-four proteins had a value below LOD in more than 20% of the samples and were filtered out.

**7. Linear mixed models (LMM) analysis**

To determine differences between allergy groups (resolution of CMA at 12 months (yes/no)) within visits, and between visits within allergy groups, an LMM with allergy status, visit and the interaction of allergy status and visit as fixed effects and subject as random effect was fit for each protein. A post-hoc test was performed to enable pairwise comparisons. A similar mixed model analysis was performed to determine differences between treatment groups (AAF/AAF-syn) within visits, and between visits within treatment groups. The fixed effects were treatment, visit and the interaction of treatment and visit, and the random effect was subject. In all cases, p-values were adjusted for multiple hypothesis testing using the Benjamini-Hochberg procedure^1^, and results with adjusted p-value ≤ 0.05 were considered significant.

**8. Pathway analysis with ConsensusPathDB**

For each protein, the mean measurement value of the samples for each of the two groups in the comparison was supplied as input. Subsequently, a paired Wilcoxon signed-rank test was carried out on each Kyoto Encyclopedia of Genes and Genomes (KEGG) pathway that included a minimum of 4 of the measured proteins. A p-value per KEGG pathway was assigned, and the p-values were corrected for multiple testing using the false discovery rate (FDR) method. The adjusted p-values are presented as q-values in the results tables, results with q-value ≤ 0.05 were considered significant.

**S1 Table.** Clinical characteristics at enrolment. P-values for differences between groups were determined by a Fisher’s exact test.

|  | **Persistent CMA at 12M**  **(n = 15)** | **Resolved CMA at 12M**  **(n = 24)** | **Total**  **(n = 39)** | **p-value** |
| --- | --- | --- | --- | --- |
| diagnosis  anaphylaxis history  DBPCFC  Open milk challenge | 0  2  13 | 2  5  17 | 2  7  30 | **0.583** |
| atopic dermatitis  mild (SCORAD < 20)  moderate (SCORAD 20-40)  severe (SCORAD > 40) | 10  4  1 | 20  2  2 | 30  6  3 | **0.257** |
|  | **AAF**  **(n = 16)** | **AAF-syn**  **(n = 23)** | **Total**  **(n = 39)** | **p-value** |
| diagnosis  anaphylaxis history  DBPCFC  Open milk challenge | 1  5  10 | 1  2  20 | 2  7  30 | **0.179** |
| atopic dermatitis  mild (SCORAD < 20)  moderate (SCORAD 20-40)  severe (SCORAD > 40) | 13  1  2 | 17  5  1 | 30  6  3 | **0.273** |

**S2 Table.** Baseline characteristics per allergy group. Numeric variables are presented as mean ± standard deviation, categorical variables are presented as number. P-values were determined by a two-sided MannWhitney U-test for numeric variables and a Fisher’s exact test for categorical variables. When the p-value was already calculated previously, this is indicated in the column “reference”. Significant differences between allergy groups are indicated in bold. Abbreviations: CM: cow’s milk, SPT: skin prick test, AD: atopic dermatitis, SCORAD: SCORing Atopic Dermatitis.

|  | **Persistent CMA at 12M**  **(n = 15)** | **Resolved CMA at 12M**  **(n = 24)** | **p-value** | **reference** |
| --- | --- | --- | --- | --- |
| age (months) | 9.68 ± 2.63 | 8.56 ± 30.04 | 0.254 | ^2^ |
| sex  female  male | 3  12 | 8  16 | 0.477 | ^2^ |
| race  Caucasian/white  Asian  other | 4  10  1 | 5  18  1 | 0.617 | this study |
| CM-specific IgE (kU/L) | 23.28 ± 50.99 | 1.70 ± 2.80 | **< 0.001** | this study |
| CM SPT wheal size (mm) | 6.80 ± 2.65 | 4.17 ± 2.20 | **0.003** | this study |
| AD severity (SCORAD) | 16.27 ± 13.24 | 8.98 ± 14.41 | **0.036** | ^2^ |
| other food allergy  yes  no | 9  6 | 10  14 | 0.333 | this study |
| treatment  AAF  AAF-syn | 6  9 | 10  14 | 1.000 | ^2^ |

**S3 Table.** Mean ± standard deviation for IgE-specific α-lactalbumin, β-lactoglobulin and casein for all infants, infants with persistent CMA and infants with resolved CMA. P-value obtained by comparing the two allergy groups with two sided Mann-Whitney U-test. Significant results are indicated in bold.

| **parameter** | **Total (n = 39)** | **Persistent CMA at visit 12M (n=15)** | **Resolved CMA at visit 12M (n=24)** | **p-value**  **(two-sided Mann-Whitney U-test)** |
| --- | --- | --- | --- | --- |
| Alpha-lactalbumin-specific IgE (kU/L)  baseline  12 months | 1.33 ± 2.62  1.06 ± 2.02 | 2.11 ± 2.76  2.25 ± 2.81 | 0.84 ± 2.46  0.31 ± 0.67 | **0.01672**  **0.00113** |
| Beta-lactoglobulin-specific IgE (kU/L)  baseline  12 months | 2.36 ± 5.06  2.07 ± 4.99 | 5.15 ± 7.36  4.98 ± 7.26 | 0.62 ± 1.03  0.26 ± 0.40 | **0.00693**  **0.00085** |
| Casein-specific IgE (kU/L)  baseline  12 months | 12.28 ± 56,85  6.66 ± 17.74 | 30.66 ± 90.45  16.74 ± 26.01 | 0.80 ± 1.44  0.35 ± 0.66 | **0.00231**  **0.00012** |

**S4 Table.** List of ethics committees, institutional review boards and regulatory authorities that approved this study.

| **Country** | **Site** | **Ethics committees, institutional review boards and regulatory authorities** | **document number** |
| --- | --- | --- | --- |
| United Kingdom | Southampton General Hospital | NRES Committee North East - Sunderland (Central Ethics Committee MREC) | 13/NE/0125 |
| Germany | Charité Hospital Berlin  St.-Marien Hospital  Ruhr-Universität Bochum im St. Josef-Hospital | Ethikkommission Charité – Ethikausschuss 2 am Campus  Virchow Klinikum Ethikkommission Ärztekammer Nordrhein Düsseldorf  Ethik-Kommission der Medizinischen Fakultät der Ruhr Universität Bochum | EA2/063/13  2013119  4679-13 |
| Italy | University Hospital Verona | Comitato Etico per la Sperimentazione Clinica della Province di Verona e Rovigo | N. prog. 2321 |
| Singapore | KK Women’s & Children’s Hospital | Singhealth Centralised Institutional Review Board (CIRB) | 2012/943/E |
| Thailand | King Chulalongkorn Memorial Hospital  Ramathibodi Hospital  Prince of Songkla Hospital | Institutional Review Board of the Faculty of Medicine, Chulalongkorn University  Committee on Human Rights Related to Research Involving Human Subjects, Faculty of Medicine Ramathibodi Hospital, Mahidol University  Ethics Committee of the Faculty of Medicine, Prince of Songkla University | COA No. 00512013, IRB No. 505/55  MURA2012/569  56-071-01-1-1 |
| United States of America | Texas Children’s Hospital | Institutional Review Board for Human Subject Research for Baylor College of Medicine and Affiliated Hospitals (BCM IRB) | H-30791 |

**S5 Table.** List of proteins after filtering out proteins that had a value below the limit of detection in more than 20% of the samples.

| **Uniprot ID** | **Olink ID** | **Symbol** | **Protein name** |
| --- | --- | --- | --- |
| Q13541 | OID00536 | 4E-BP1 | Eukaryotic translation initiation factor 4E-binding protein 1 |
| P00813 | OID00560 | ADA | Adenosine Deaminase |
| Q14790 | OID00550 | CASP-8 | Caspase-8 |
| Q99731 | OID00513 | CCL19 | C-C motif chemokine 19 |
| P78556 | OID00556 | CCL20 | C-C motif chemokine 20 |
| P55773 | OID00530 | CCL23 | C-C motif chemokine 23 |
| Q9NRJ3 | OID00539 | CCL28 | C-C motif chemokine 28 |
| P10147 | OID00532 | CCL3 | C-C motif chemokine 3 |
| P13236 | OID00498 | CCL4 | C-C motif chemokine 4 |
| P25942 | OID00542 | CD40 | CD40L receptor |
| P06127 | OID00531 | CD5 | T-cell surface glycoprotein CD5 |
| Q9H5V8 | OID00476 | CDCP1 | CUB domain-containing protein 1 |
| P09603 | OID00562 | CSF-1 | Macrophage colony-stimulating factor 1 |
| P28325 | OID00491 | CST5 | Cystatin D |
| P78423 | OID00552 | CX3CL1 | Fractalkine |
| P09341 | OID00496 | CXCL1 | C-X-C motif chemokine 1 |
| P02778 | OID00535 | CXCL10 | C-X-C motif chemokine 10 |
| O14625 | OID00486 | CXCL11 | C-X-C motif chemokine 11 |
| P42830 | OID00520 | CXCL5 | C-X-C motif chemokine 5 |
| P80162 | OID00534 | CXCL6 | C-X-C motif chemokine 6 |
| Q07325 | OID00490 | CXCL9 | C-X-C motif chemokine 9 |
| Q8NFT8 | OID01213 | DNER | Delta and Notch-like epidermal growth factor-related  receptor |
| P80511 | OID00541 | EN-RAGE | Protein S100-A12 |
| O95750 | OID00545 | FGF-19 | Fibroblast growth factor 19 |
| P49771 | OID00533 | Flt3L | Fms-related tyrosine kinase 3 ligand |
| P14210 | OID00522 | HGF | Hepatocyte growth factor |
| P01583 | OID00493 | IL-1 alpha | Interleukin-1 alpha |
| Q08334 | OID00515 | IL-10RB | Interleukin-10 receptor subunit beta |
| P29460 | OID00523 | IL-12B | Interleukin-12 subunit beta |
| Q13261 | OID00514 | IL-15RA | Interleukin-15 receptor subunit alpha |
| Q14116 | OID00501 | IL-18 | Interleukin-18 |
| Q13478 | OID00517 | IL-18R1 | Interleukin-18 receptor 1 |
| Q9UHF4 | OID00489 | IL-20RA | Interleukin-20 receptor subunit alpha |
| P05231 | OID00482 | IL-6 | Interleukin-6 |
| P13232 | OID00478 | IL-7 | Interleukin-7 |
| P10145 | OID00471 | IL-8 | Interleukin-8 |
| P01137 | OID00480 | LAP TGF-beta-1 | Latency-associated peptide transforming growth factor  beta-1 |
| P15018 | OID00547 | LIF | Leukemia inhibitory factor |
| P42702 | OID00511 | LIF-R | Leukemia inhibitory factor receptor |
| P13500 | OID00484 | MCP-1 | Monocyte chemotactic protein 1 |
| P80075 | OID00549 | MCP-2 | Monocyte chemotactic protein 2 |

**S5 Table.** (continued)

| **Uniprot ID** | **Olink ID** | **Symbol** | **Protein name** |
| --- | --- | --- | --- |
| Q99616 | OID00504 | MCP-4 | Monocyte chemotactic protein 4 |
| P03956 | OID00510 | MMP-1 | Matrix metalloproteinase-1 |
| P09238 | OID00527 | MMP-10 | Matrix metalloproteinase-10 |
| O00300 | OID00479 | OPG | Osteoprotegerin |
| P13725 | OID00494 | OSM | Oncostatin-M |
| Q9NZQ7 | OID00518 | PD-L1 | Programmed cell death 1 ligand 1 |
| P21583 | OID00500 | SCF | Stem cell factor |
| Q8IXJ6 | OID00538 | SIRT2 | SIR2-like protein 2 |
| O95630 | OID00558 | STAMBP | STAM-binding protein |
| P01135 | OID00503 | TGF-alpha | Transforming growth factor alpha |
| P01375 | OID05548 | TNF | Tumor necrosis factor |
| Q07011 | OID00553 | TNFRSF9 | Tumor necrosis factor receptor superfamily member 9 |
| O43557 | OID00506 | TNFSF14 | Tumor necrosis factor ligand superfamily member 14 |
| P50591 | OID00488 | TRAIL | TNF-related apoptosis-inducing ligand |
| O43508 | OID00555 | TWEAK | Tumor necrosis factor (Ligand) superfamily, member 12 |
| P00749 | OID00481 | uPA | Urokinase-type plasminogen activator |
| P15692 | OID00472 | VEGFA | Vascular endothelial growth factor A |

**S6 Table.** Results of comparing visits within allergy groups using linear mixed models and post hoc analysis. Allergic = persistent CMA at visit 12M; tolerant = resolved CMA at visit 12M. Number of samples: persistent CMA – 0M: 15, persistent CMA – 6M: 15, persistent CMA – 12M: 14, resolved CMA – OM: 24, resolved CMA – 6M: 24, resolved CMA – 12M: 24.

See Excel file S6_Table.xlsx

**S7 Table.** Results of comparing allergy groups within visits using linear mixed models and post hoc analysis. Allergic = persistent CMA at visit 12M; tolerant = resolved CMA at visit 12M. Number of samples: persistent CMA – 0M: 15, persistent CMA – 6M: 15, persistent CMA – 12M: 14, resolved CMA – OM: 24, resolved CMA – 6M: 24, resolved CMA – 12M: 24.

See Excel file S7_Table.xlsx

**S8 Table.** Results of comparing visits within treatment groups using linear mixed models and post hoc analysis. AAF: standard amino acid formula; AAF-syn: amino acid formula with synbiotic blend. Number of samples: AAF – OM: 16, AAF – 6M: 16, AAF-12M: 16, AAF-syn – 0M: 23, AAF-syn – 6M: 23, AAF-syn – 12M: 22.

See Excel file S8_Table.xlsx

**S9 Table.** Results of comparing treatment groups within visits using linear mixed models and post hoc analysis. AAF: standard amino acid formula; AAF-syn: amino acid formula with synbiotic blend. Number of samples: AAF – OM: 16, AAF – 6M: 16, AAF-12M: 16, AAF-syn – 0M: 23, AAF-syn – 6M: 23, AAF-syn – 12M: 22.

See Excel file S9_Table.xlsx

**S10 Table.** Results of comparing visits within allergy groups using KEGG pathway enrichment in ConsensusPathDB. Pathways with at least 4 measured proteins are displayed. Allergic = persistent CMA at visit 12M; tolerant = resolved CMA at visit 12M. Number of samples: persistent CMA – 0M: 15, persistent CMA – 6M: 15, persistent CMA – 12M: 14, resolved CMA – OM: 24, resolved CMA – 6M: 24, resolved CMA – 12M: 24.

See Excel file S10_Table.xlsx

**S11 Table.** Results of comparing allergy groups within visits using KEGG pathway enrichment in ConsensusPathDB. Pathways with at least 4 measured proteins are displayed. Allergic = persistent CMA at visit 12M; tolerant = resolved CMA at visit 12M. Number of samples: persistent CMA – 0M: 15, persistent CMA – 6M: 15, persistent CMA – 12M: 14, resolved CMA – OM: 24, resolved CMA – 6M: 24, resolved CMA – 12M: 24.

See Excel file S11_Table.xlsx

**S12 Table.** Results of comparing visits within treatment groups using KEGG pathway enrichment in ConsensusPathDB. Pathways with at least 4 measured proteins are displayed. AAF: standard amino acid formula; AAF-syn: amino acid formula with synbiotic blend. Number of samples: AAF – OM: 16, AAF – 6M: 16, AAF-12M: 16, AAF-syn – 0M: 23, AAF-syn – 6M: 23, AAF-syn – 12M: 22.

See Excel file S12_Table.xlsx

**S13 Table.** Results of comparing treatment groups within visits using KEGG pathway enrichment in ConsensusPathDB. Pathways with at least 4 measured proteins are displayed. AAF: standard amino acid formula; AAF-syn: amino acid formula with synbiotic blend. Number of samples: AAF – OM: 16, AAF – 6M: 16, AAF-12M: 16, AAF-syn – 0M: 23, AAF-syn – 6M: 23, AAF-syn – 12M: 22.

See Excel file S13_Table.xlsx

**S14 Table.** Comparison of the number of infections (num inf). (A) Between 6-month visit intervals within allergy groups. P-values were determined by a paired two-sided MannWhitney U-test. (B) Between allergy groups within 6-month visit intervals. P-values were determined by a two-sided MannWhitney U-test. (C) Between 6-month visit intervals within treatment groups. P-values were determined by a paired two-sided MannWhitney U-test. (D) Between treatment groups within 6-month visit intervals. P-values were determined by a two-sided MannWhitney U-test.

|  | comparison | | results | | |
| --- | --- | --- | --- | --- | --- |
|  | 1 | 2 | mean1 ± sd1 | mean2 ± sd2 | p-value |
| (A) | persistent CMA at 12M  num inf 0 to 6M | persistent CMA at 12M  num inf 6 to 12M | 1.67 ± 1.45 | 1.33 ± 1.23 | 0.440 |
|  | resolved CMA at 12M  num inf 0 to 6M | resolved CMA at 12M  num inf 6 to 12M | 1.25 ± 4.61 | 0.71 ± 0.81 | 0.079 |
| (B) | persistent CMA at 12M  num inf 0 to 6M | resolved CMA at 12M  num inf 0 to 6M | 1.67 ± 1.45 | 1.25 ± 4.61 | 0.176 |
|  | persistent CMA at 12M  num inf 6 to 12M | resolved CMA at 12M  num inf 6 to 12M | 1.33 ± 1.23 | 0.71 ± 0.81 | 0.108 |
| (C) | AAF  num inf 0 to 6M | AAF  num inf 6 to 12M | 1.56 ± 1.71 | 1.06 ± 1.18 | 0.192 |
|  | AAF-syn  num inf 0 to 6M | AAF-syn  num inf 6 to 12M | 1.30 ± 1.52 | 0.87 ± 0.92 | 0.177 |
| (D) | AAF  num inf 0 to 6M | AAF-syn  num inf 0 to 6M | 1.56 ± 1.71 | 1.30 ± 1.52 | 0.688 |
|  | AAF  num inf 6 to 12M | AAF-syn  num inf 6 to 12M | 1.06 ± 1.18 | 0.87 ± 0.92 | 0.739 |

**S1 Fig.** Flowchart of the subject selection process. Red boxes: selection within the PRESTO clinical trial^3^, blue boxes: selection within the Earlyfit project^2^. The final aim of the Earlyfit project is to combine data from different omics/platforms into a predictive model. Therefore samples for different omics/platforms are taken from the same subjects.

**
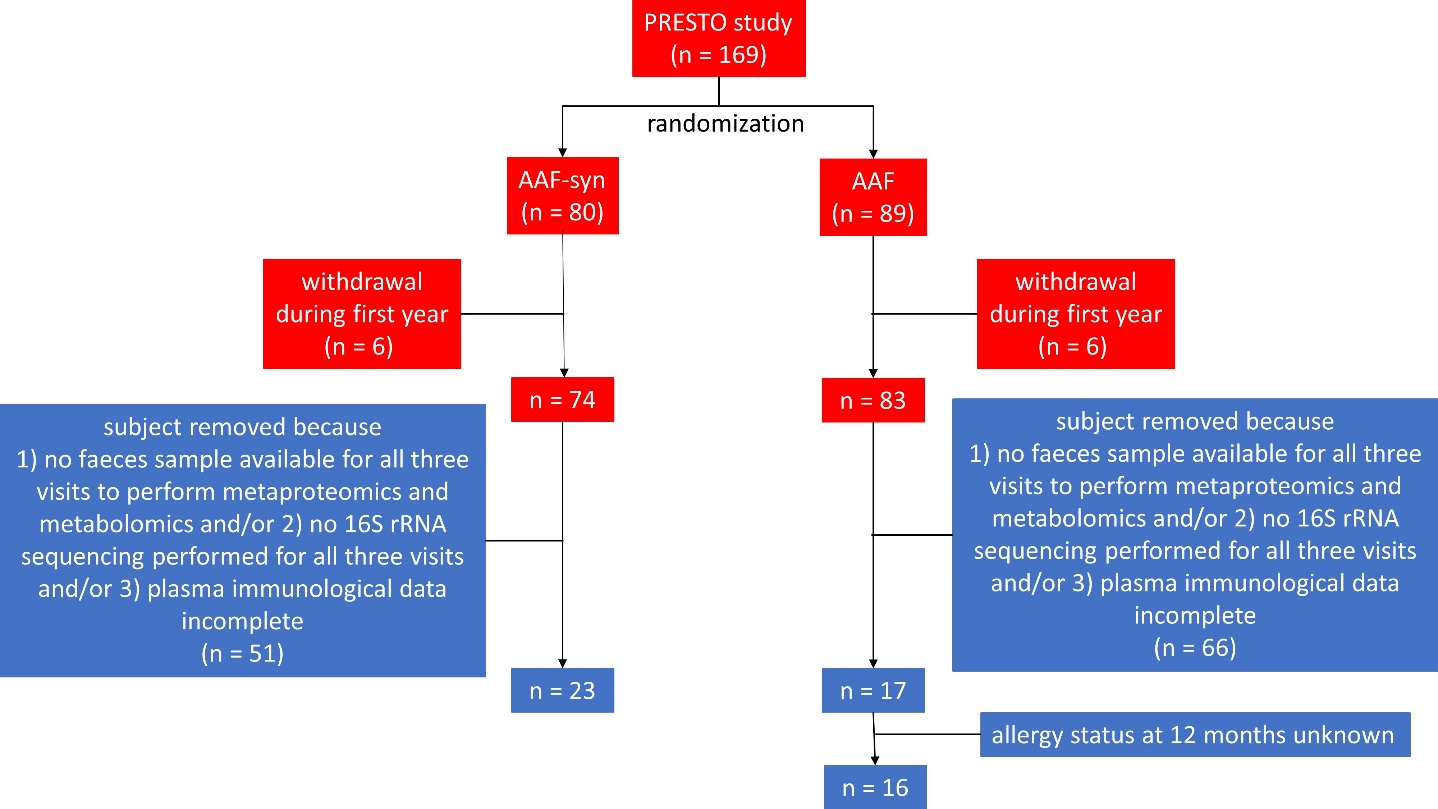
**

**S2 Fig.** Principal components analysis colored by QC warning and showing the percentage of explained variance by PC1 and PC2.


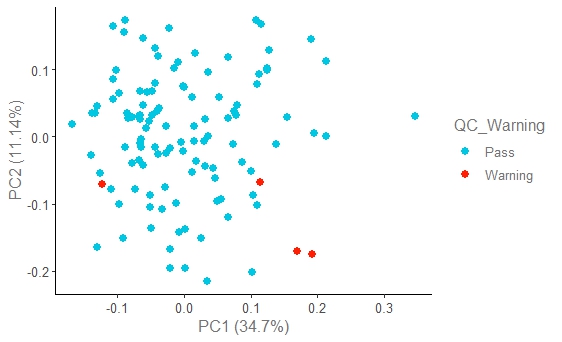


**S3 Fig.** Boxplots of NPX values for the proteins with significant differences between visits within allergy groups as determined by linear mixed models and post hoc analysis. n.s: not significant (adjusted p-value > 0.05), *: 0.01 < adjusted p-value ≤ 0.05, **: 0.005 < adjusted p-value ≤ 0.01, ***: adjusted p-value ≤ 0.005.


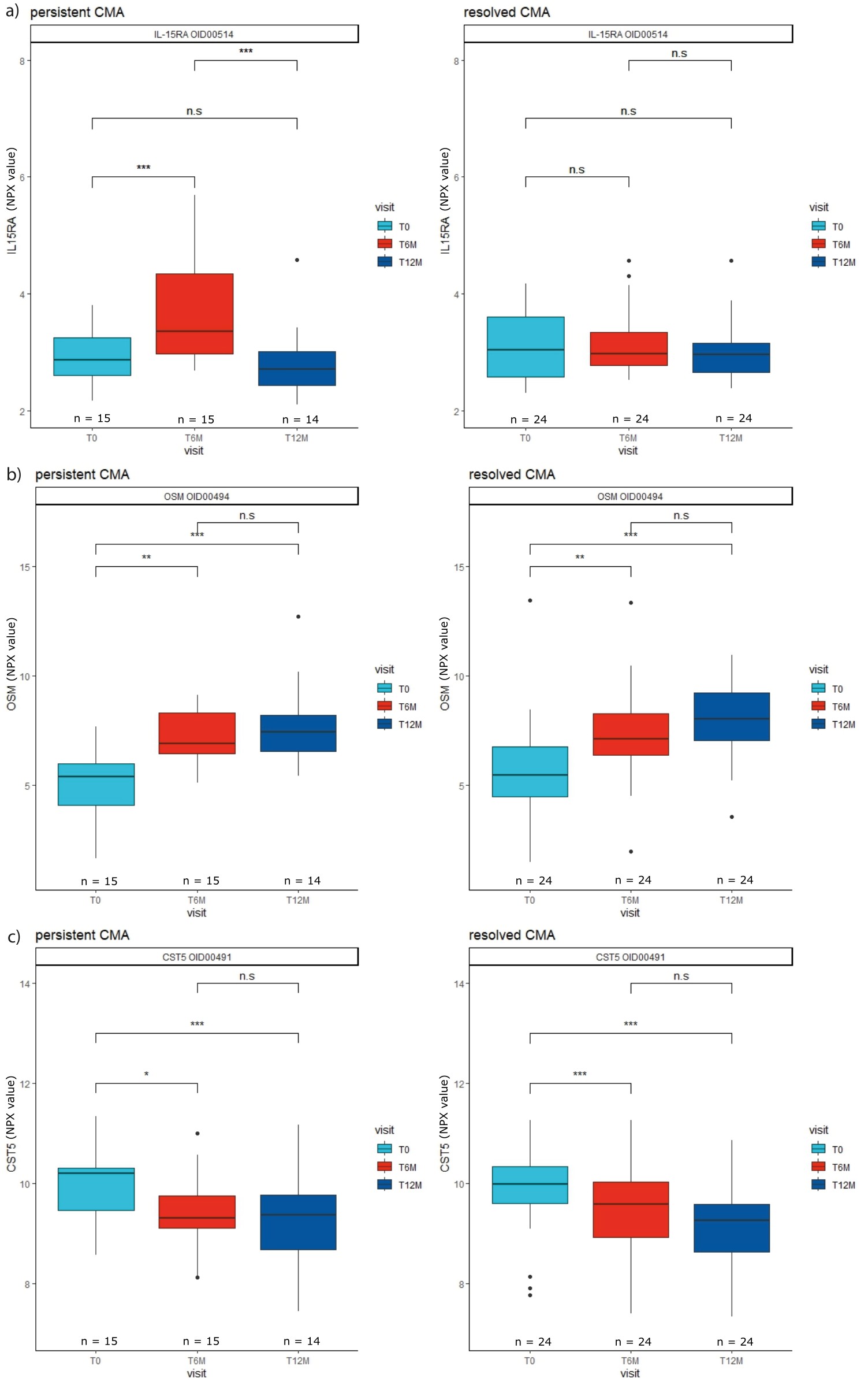


**S3 Fig.** (continued)


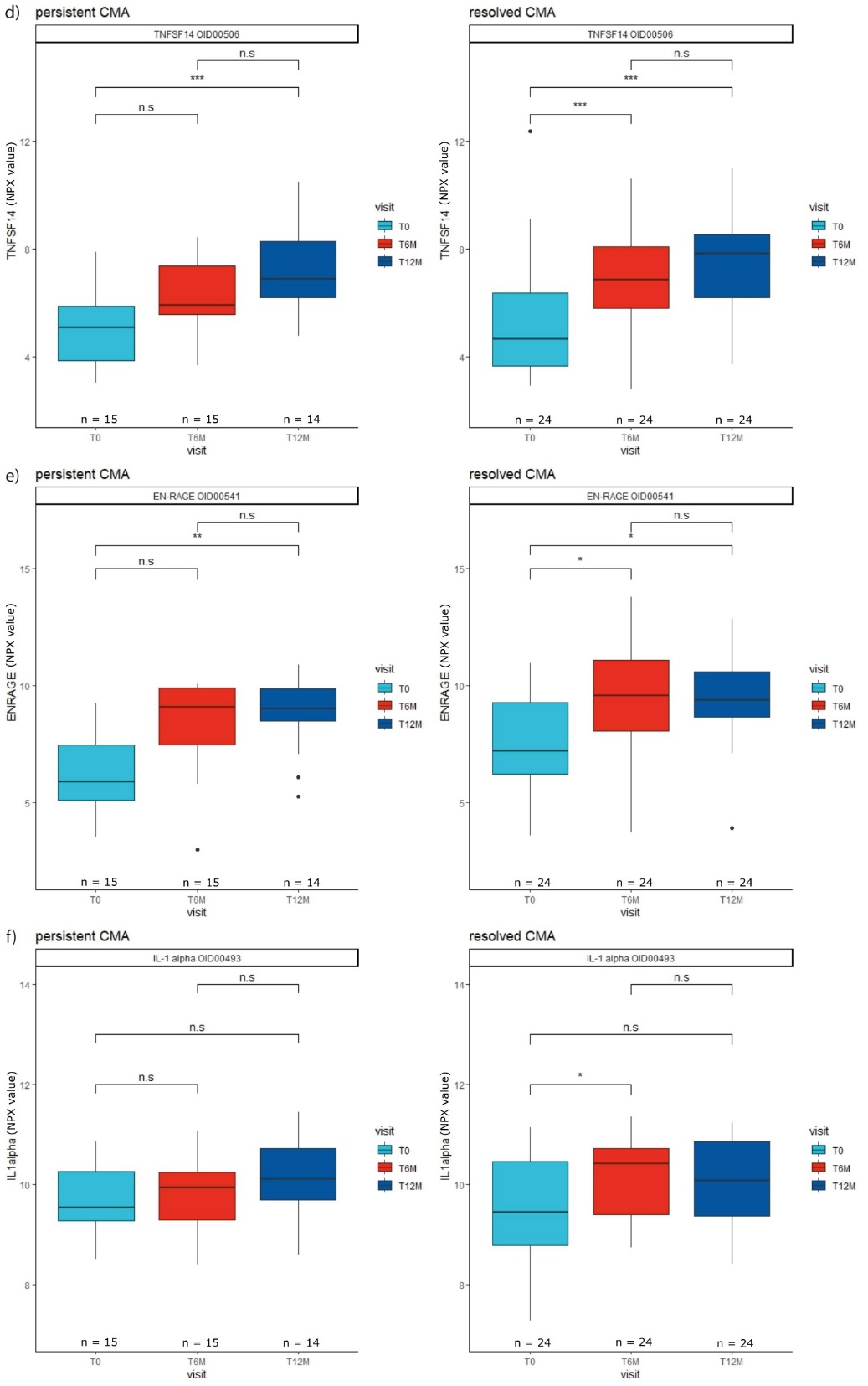


**S3 Fig.** (continued)


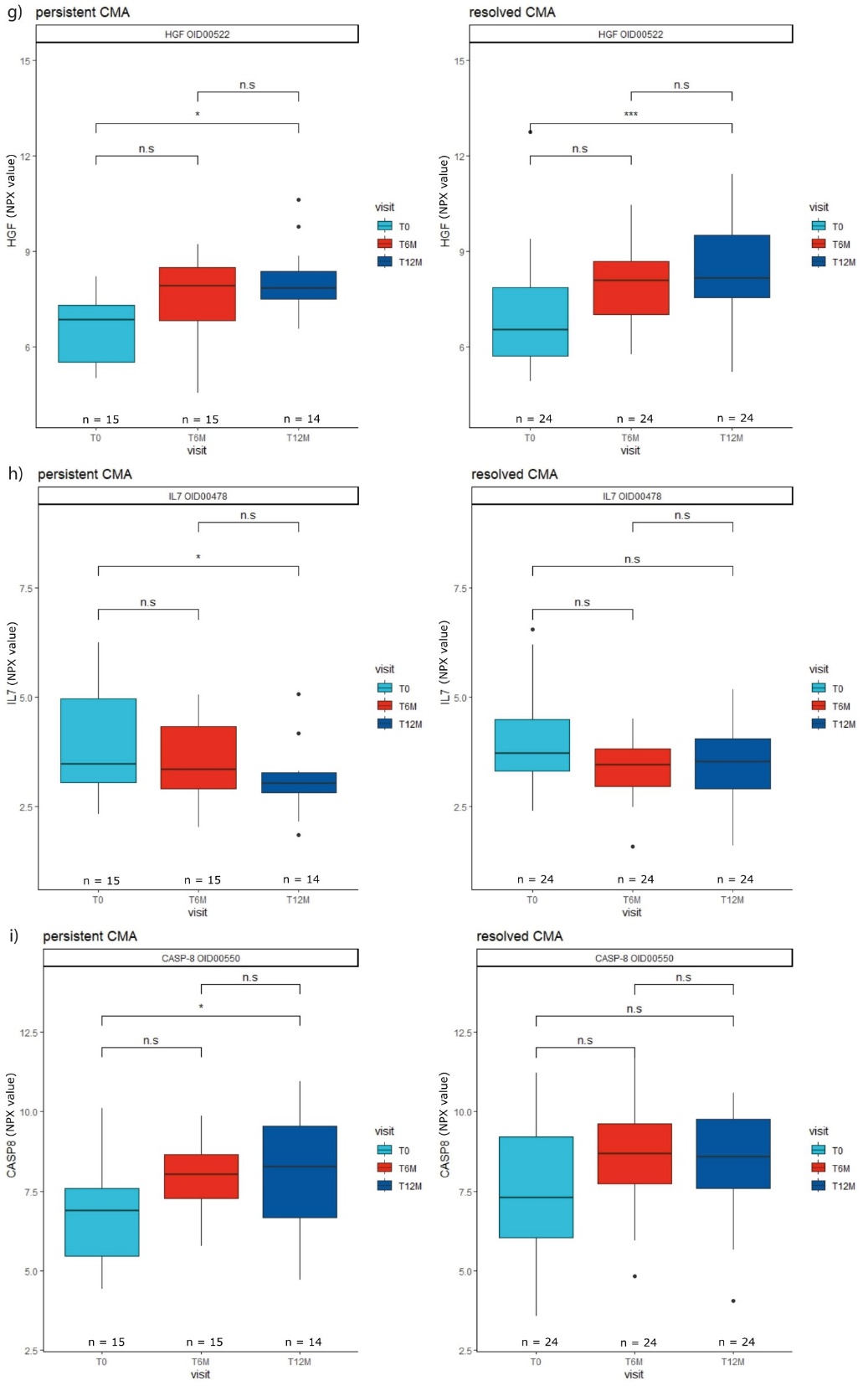


**S3 Fig.** (continued)


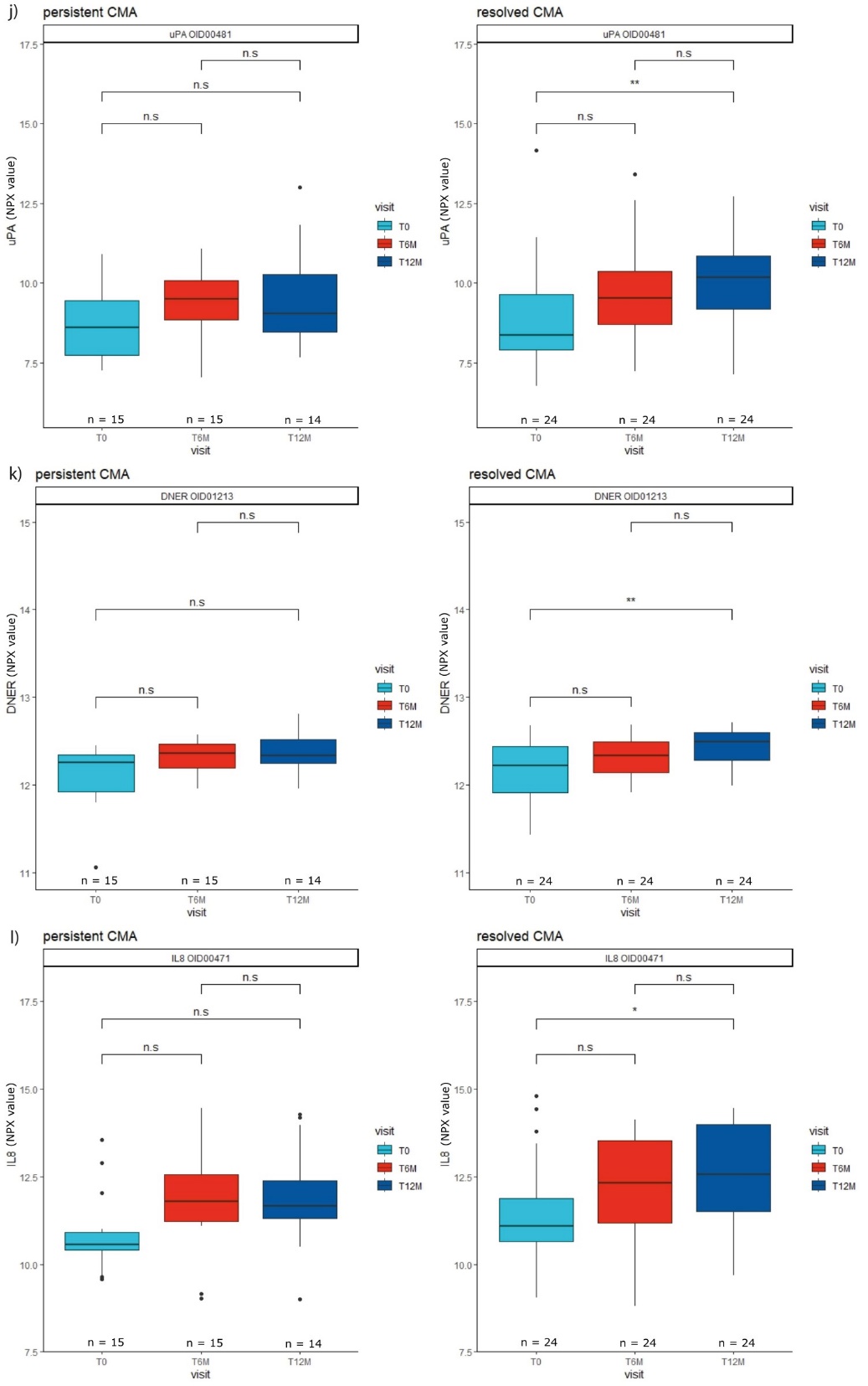


**S3 Fig.** (continued)


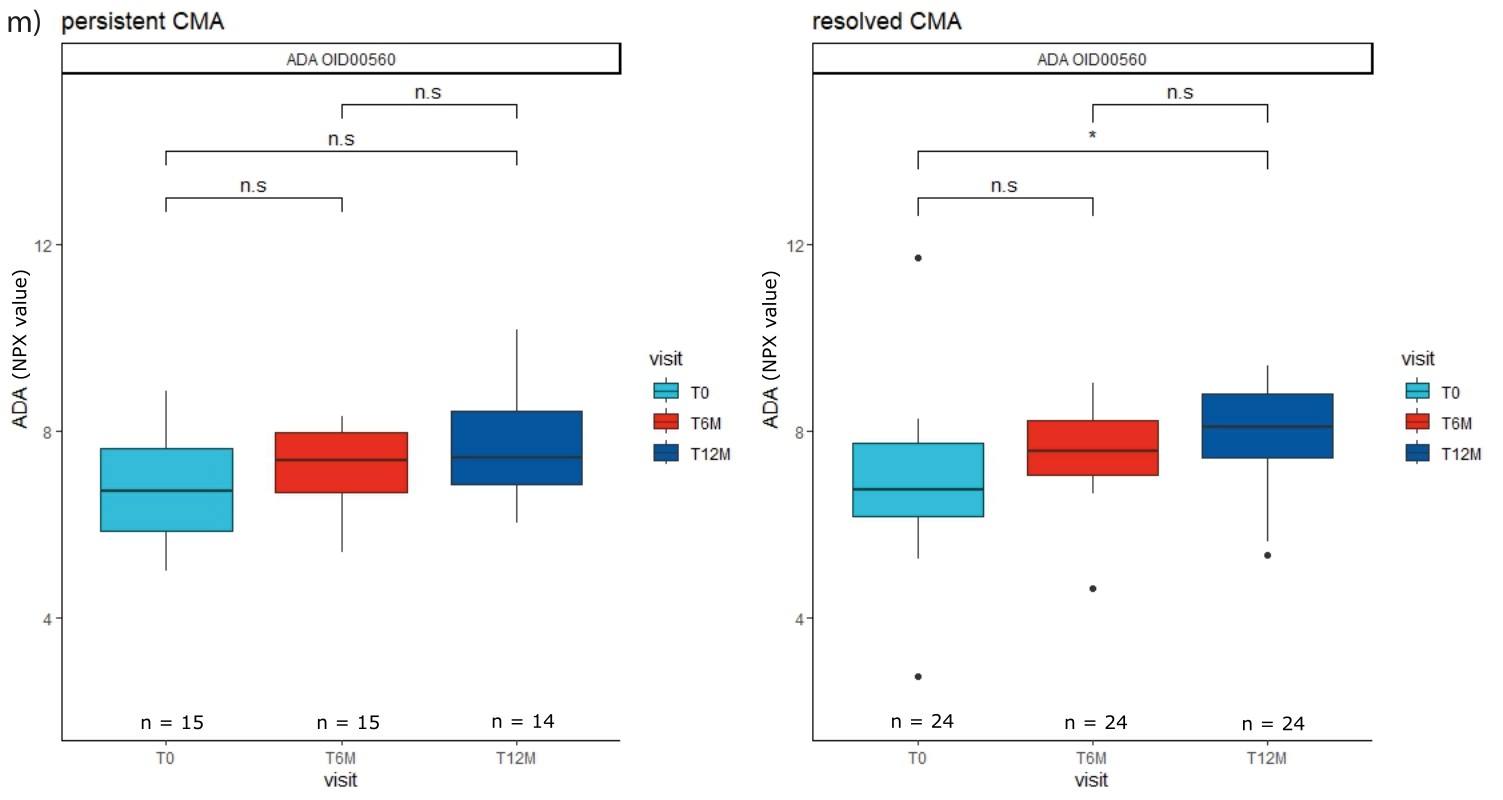


**S4 Fig.** Boxplots of NPX values for the proteins with significant differences between visits within treatment groups as determined by linear mixed models and post hoc analysis. n.s: not significant (adjusted p-value > 0.05), *: 0.01 < adjusted p-value ≤ 0.05, **: 0.005 < adjusted p-value ≤ 0.01, ***: adjusted p-value ≤ 0.005.


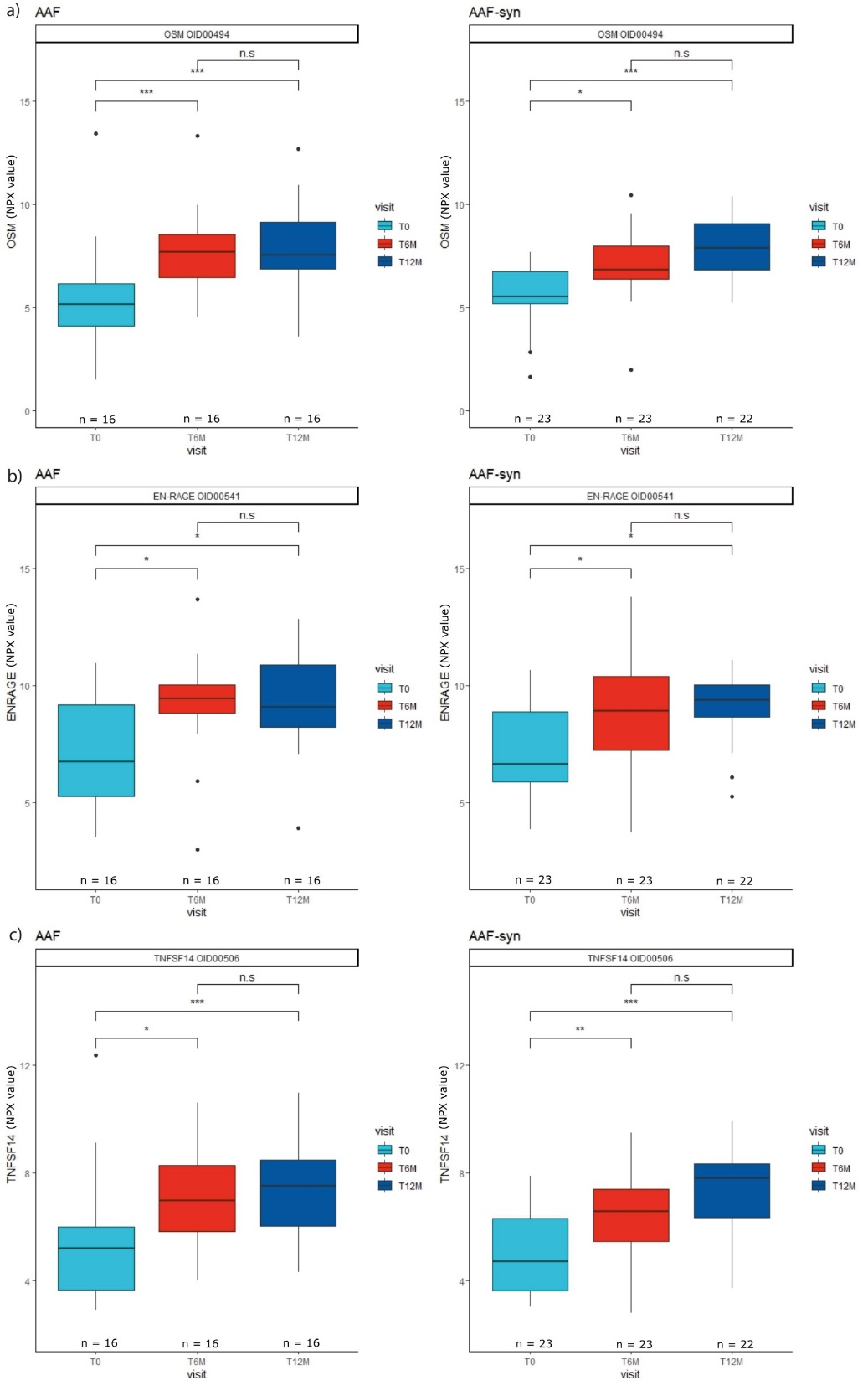


**S4 Fig.** (continued)


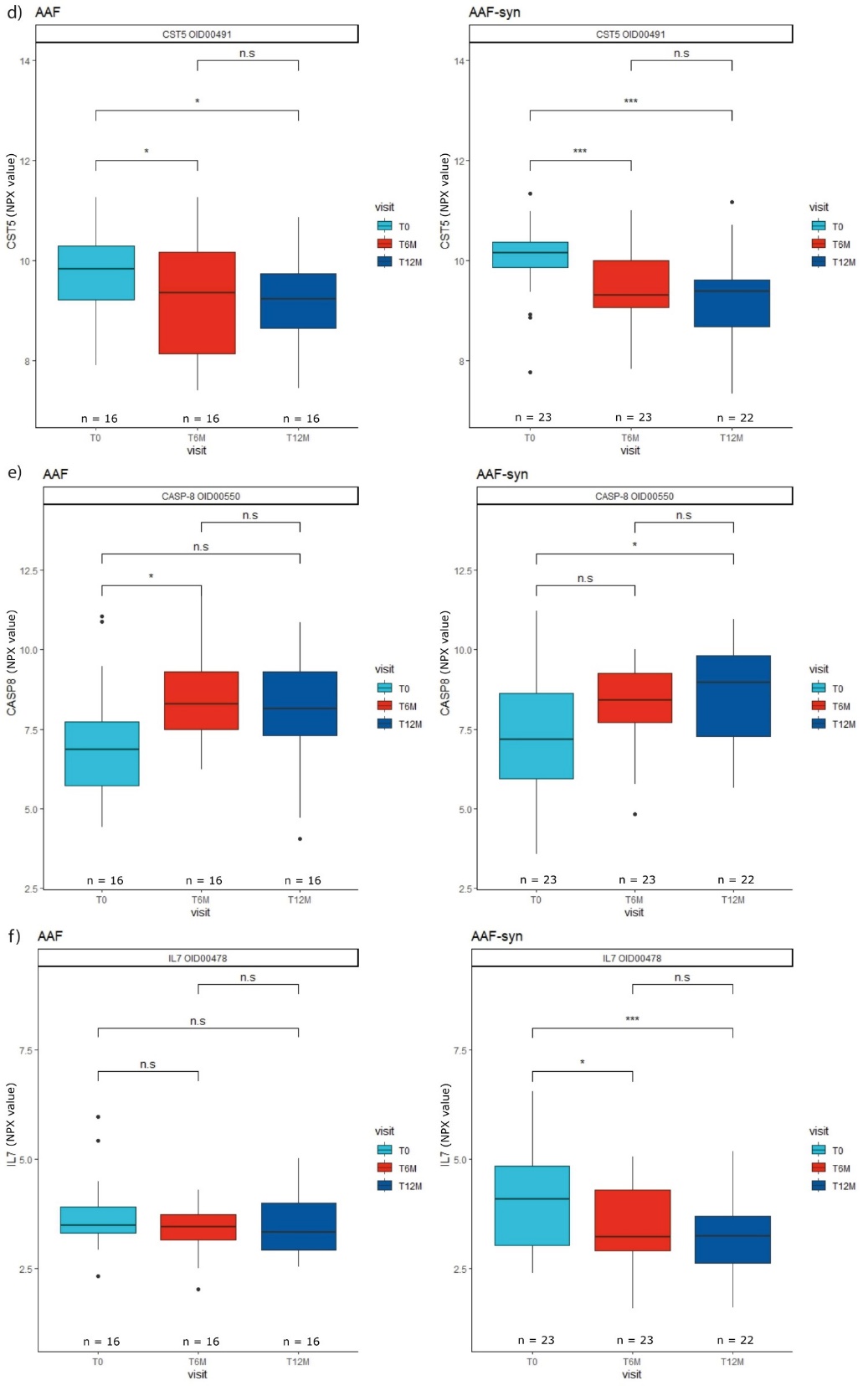


**S4 Fig.** (continued)


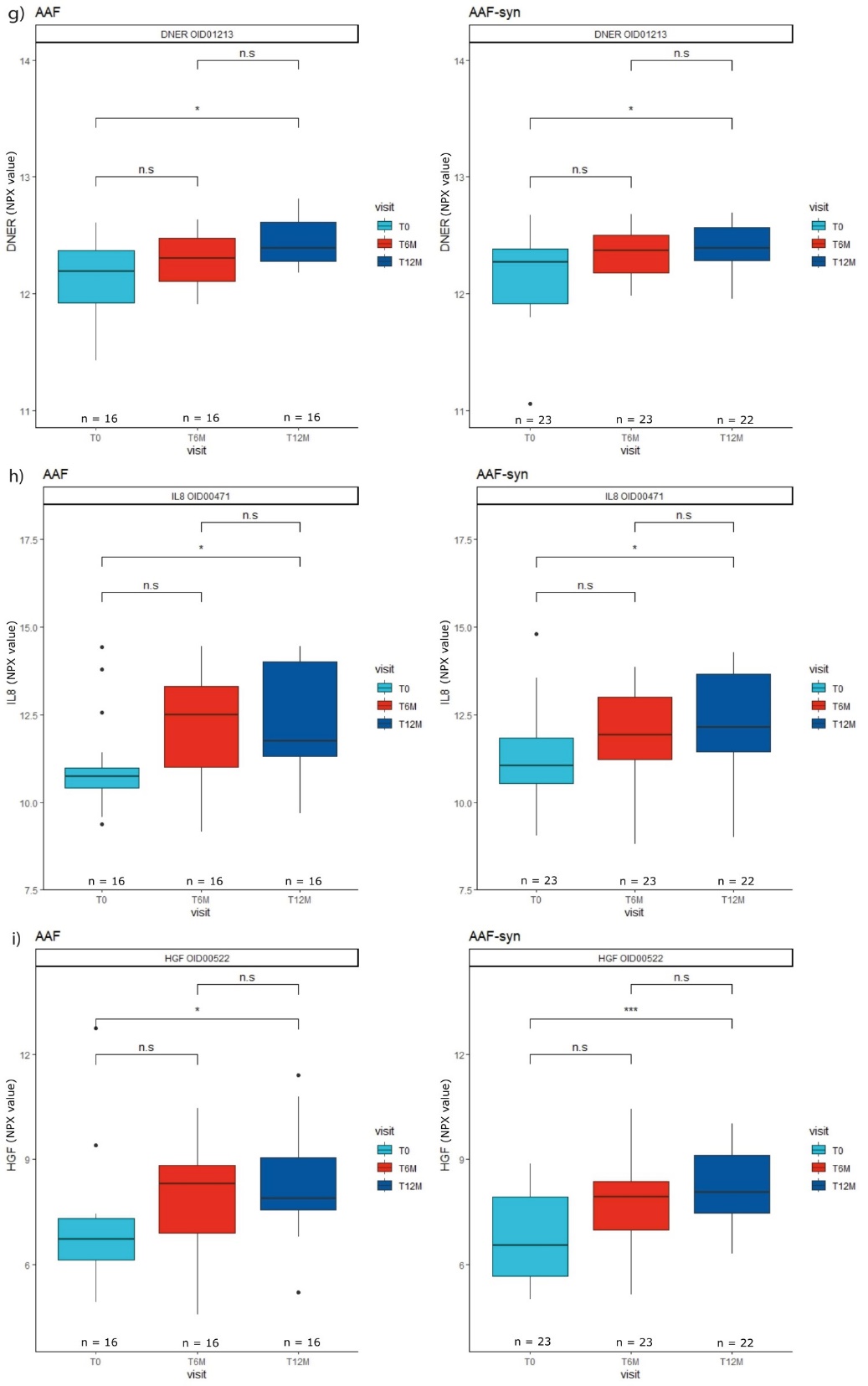


**S4 Fig.** (continued)


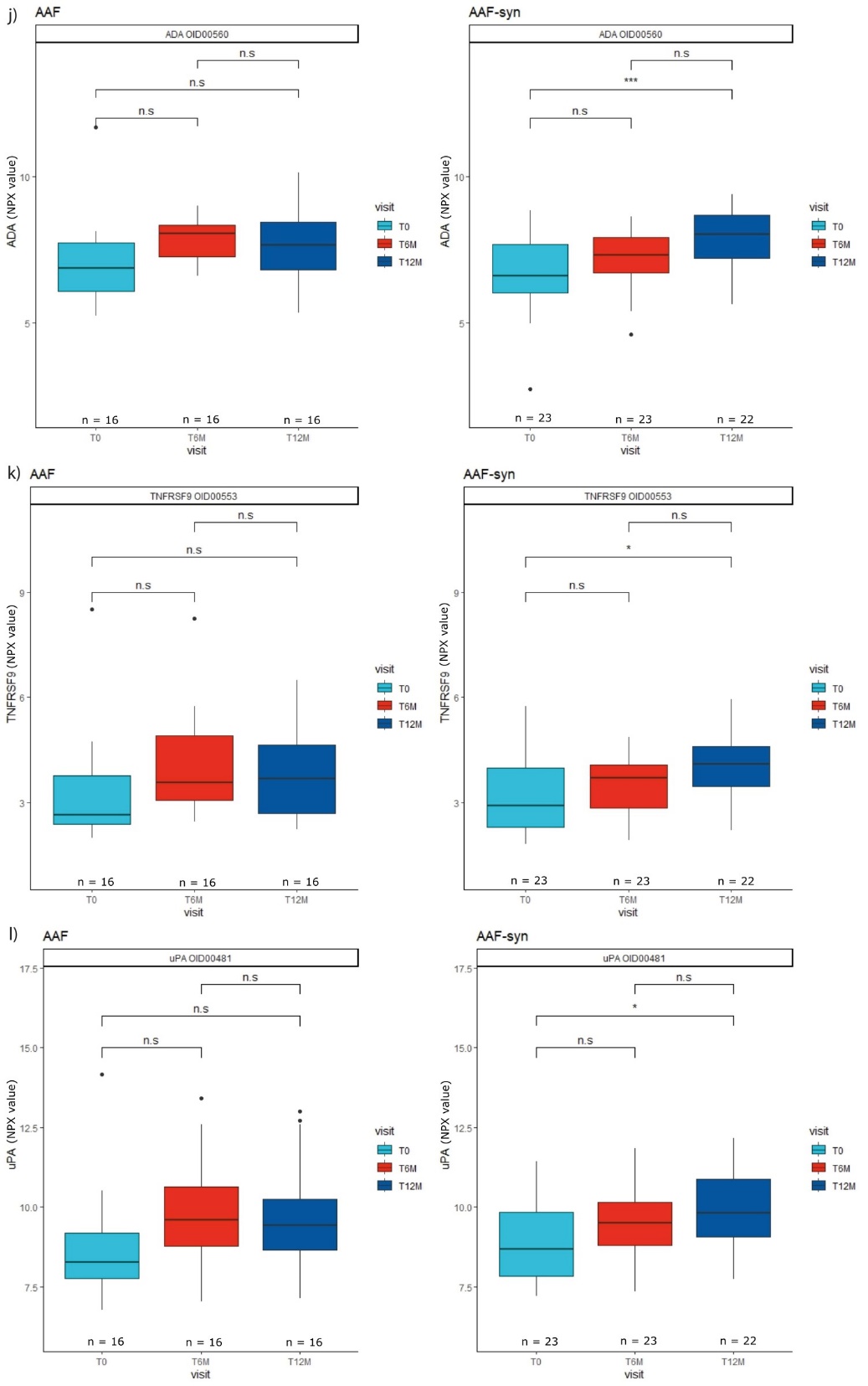


**S4 Fig.** (continued)


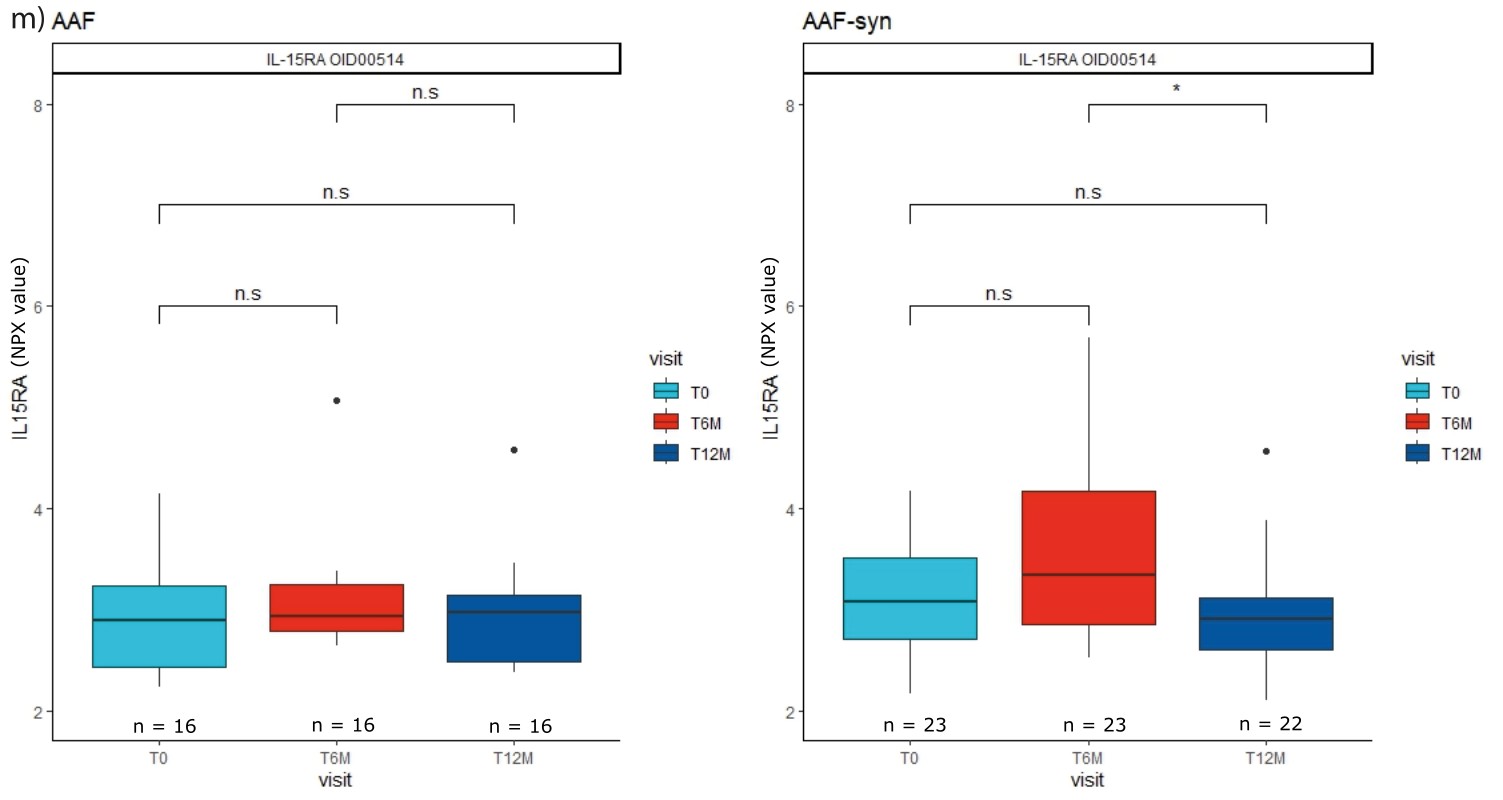


1. Benjamini Y, Hochberg Y. Controlling the False Discovery Rate: A Practical and Powerful Approach to Multiple Testing. *Journal of the Royal Statistical Society: Series B (Methodological)*. 1995;57(1):289-300. doi:10.1111/j.2517-6161.1995.tb02031.x

2. Hendrickx DM, An R, Boeren S, et al. Assessment of infant outgrowth of cow’s milk allergy in relation to the faecal microbiome and metaproteome. *Sci Rep*. 2023;13(1):12029. doi:10.1038/s41598-023-39260-w

3. Chatchatee P, Nowak-Wegrzyn A, Lange L, et al. Tolerance development in cow’s milk–allergic infants receiving amino acid–based formula: A randomized controlled trial. *Journal of Allergy and Clinical Immunology*. 2022;149(2):650-658.e5. doi:10.1016/j.jaci.2021.06.025
