# Supplementary material for: Identification of potential inflammation markers for outgrowth of cow’s milk allergy
Source: PLoS One. 2025 Sep 10;20(9):e0331462. doi: 10.1371/journal.pone.0331462 (PMC12422486; doi:10.1371/journal.pone.0331462)
Supplement: Attachment — Submitted filename: response_to_reviewers.pdf [file pone.0331462.s011.pdf]

Dear Editor,

We thank you and the reviewers for the efficient handling of the manuscript and the valuable comments provided. Below we address these comments in a point-by-point manner and explain the corresponding changes in the revised manuscript (changes in the updated manuscript are highlighted in red colour).

Kind regards,
Dr. Clara Belzer
(on behalf of all co-authors)

Reviewer #1: I read with great interest the manuscript "Supplementary Material for: Identification of potential inflammation markers for outgrowth of cow's milk allergy".The topic is of interest; however many criticisms rise regarding the study design and the inclusion criteria. My comments, as following:
-Study design: Flowchart of the subject selection process (see also S1 Fig): 169 patients were randomized to the formulas (AAF-syn 80 vs AAF 89), however only 23/80 and 17/89 respectively remained after removing patients (no feces sample available, no 16S rRNA sequencing performed, plasma immunological data incomplete). This represents a main bias for the results interpretations and conclusions
We agree that the substantial reduction in sample size due to the exclusion of these participants introduces a potential bias, which may have affected the results, interpretation and conclusions.
In the first sentence of the subsection "Sample collection and experimental design" of the methods section, we added "based on sample and data availability" (line 82).
We added the following paragraph to the discussion: "The selection of only a subset of patients based on sample and data availability may have introduced selection bias, as the remaining participants may not be representative of the initial study population. Moreover, the limited sample size of our study may limit the generalizability of our findings. Therefore, the results should be considered as hypothesis-generating and need confirmation in future studies."(lines 489-493)

- Inclusion criteria: I am puzzled to see that more than 60% of subjects resolved their IgE allergy at 12 months of life. This is even higher than that observed in Europrevall Study (57% of those with confirmed CMA at 12 months), which was amdong the highest reported in literature. Most of recent powered studies evaluating the natural history of IgE mediated CMA reported a lower percentage of tolerance at 12 months of that observed in Europrevall study. This rise doubt on the criteria used to diagnose IgE cow's milk allergy (Line 59) in the PRESTO Study.
We like to clarify that the infants in our study were older than 12 months of age when they developed tolerance to CM. At baseline, they were between 3 and 13 months old (see methods section, line 129). On average, they were ca 9 months. We added the mean age at baseline in the methods (line 129). So this means that after being 12 months in the study, they were between 15 and 25 months old (mean ca 21 months). In the complete PRESTO cohort, the % of subjects that resolved their IgE allergy after being 12 months in the study was 49% and in line with natural outgrowth (see Chatchatee et al, 2022, Food allergy and gastrointestinal disease, Volume 149, Issue 2, p650-658, https://www.jacionline.org/article/S0091-6749(21)01053-8/fulltext)(see also methods section, lines 92-93). The higher percentage of infants that resolved their IgE allergy after being 12 months in the study in our subset of PRESTO is due to the selection procedure based on sample and data availability. In the discussion of the revised manuscript we indicate that the subset may not be representative of the initial study population because of the selection procedure (see answer to your previous question on study design).

Looking at Supplementary Material demonostration of IgE mediated CMA was defined as following: " infants sensitized to CM, confirmed by CM-specific serum IgE >0.1 kU/L and/or CM skin prick test wheal size ≥3 mm, were diagnosed with CMA as follows. For some infants, CMA diagnosis was confirmed by an open or double-blind placebo-controlled CM challenge (DBPCFC). For others, diagnosis was confirmed by a history of anaphylaxis reaction to CM reported by two physicians. Furthermore, looking at Table S1 we can see that severity of atopic dermatitis has been reported; thus it means that some infants had concomitant AD, and it is well known that AD

infants may be sensitized to trofoallergens, especially CM proteins but they are not real allergic to CM. This is even more true if a confirmation OFC is not performed to confirm the diagnosis of CMA. Another point of interest regards the IgE level which was the lowest which may be considered (0.1, instead of 0.35 as often used as cut off to consider IgE sensitization). The percentage of infants who underwent a confirmatory open OFC need to be explicited as well as the percentage of infants with concomitant AD. Clinical characteristics of infants at the enrollment, especially regarding symptoms at onset, are lacking also in supplementary materials should be provided (how many patients with anaphylaxis? How many with AD and so on..)All the above mentioned issues, regarding the inclusion criteria and the CMA diagnosis itself, joined to the small sample size, represent important methodological lacks, which call in questions the observed results and limit the validity of the conclusions.
We added the clinical characteristics of infants at the enrollment to the supplementary material (see S1 Table). The cut-off of 0.1 kU/L for the IgE level (instead of 0.35) was decided with the clinical investigators as a clinically relevant cutoff for determining sensitization, specifically for young children as included in PRESTO. In addition, this criterion was always applied in combination with an open food challenge or a DBPCFC, unless the intake of cow's milk resulted in an anaphylactic reaction. The combination was necessary for eligibility. We added the following to S1 File, section 1:"The criterion of CM-specific serum IgE >0.1 kU/L was always applied in combination with an open food challenge or a DBPCFC, unless the intake of cow's milk resulted in an anaphylactic reaction. The combination was necessary for eligibility."

-Last but not least, patients were fed with AAF which usually should be reserved to infants/children with more severe symptoms. Again, considering the study population, 61.5% reached tolerance at 12 months, that implies that most of patients had no severe form of CMA (if we consider "true" the diagnosis of IgE CMA and not simply sensitization).
We agree with the reviewer that symptoms were mild during enrollment in PRESTO, but that does not mean they did not have severe CMA. Multiple cow's milk elimination diets had been used frequently before enrollment. We added the following paragraph to the discussion: "The PRESTO study aimed to include infants for whom AAF is recommended. Infants were frequently on a CM elimination diet before enrollment and consequently had mild but persistent symptoms[11]."(lines 479-480)

- In the Discussion section limitations regarding the above mentioned issues are lacking (except for the small sample size)Overall, main methodological lacks hamper the initial eagerness about the scope of the manuscript.
The limitations mentioned by the reviewer are discussed in our revised paper, see my answers to the previous questions.

Reviewer #2: This study investigates the natural resolution mechanisms of cow's milk allergy by analyzing changes in multiple cytokines using saliva, a non-invasive specimen. The finding that specific cytokine trajectories differ between children with and without resolution is noteworthy and meaningful as a hypothesis-generating study. However, the study has several limitations, including a small sample size and the retrospective nature of a secondary analysis derived from an interventional trial. The authors are requested to address the following points. If any of these are considered limitations, they should be clearly acknowledged in the "Limitations" section:
#1 Do salivary cytokine levels reliably reflect systemic (blood) cytokine concentrations?
We added the following paragraph to the discussion: "Saliva is a body fluid containing proteins, cytokines and immunoglobulins, reflecting the oral/mucosal immune response. There are recent studies showing an association between cytokines found in saliva and blood plasma. Another study has compared the inflammatory profile for saliva and serum samples of IBD patients using Olink and actually showed that approximately 40% of the markers were expressed in both saliva and serum samples, however the association between serum and saliva was poor. In addition in this study it was shown that the saliva results were of additive value for monitoring disease activity".(lines 471-477)

#2 What criteria were used to select participants from the AAF and AAF-syn interventional trial for inclusion in the present analysis?

See S1 fig in S1 file. The participants were selected based on sample and availability of other types of data in function of the final aim of our project (Earlyfit): to combine data from different omics/platforms into a predictive model. Therefore participants were selected by availability of samples and other (-omics and non-omics) data types (participants with no feces sample available, no 16S rRNA sequencing performed or plasma immunological data incomplete were removed). We agree that the substantial reduction in sample size due to the exclusion of these participants introduces a potential bias, which may have affected the results, interpretation and conclusions. In the first sentence of the subsection “Sample collection and experimental design” of the methods section, we added “based on sample and data availability”.(line 82).

We added the following paragraph to the discussion: “The selection of only a subset of patients based on sample and data availability may have introduced selection bias, as the remaining participants may not be representative of the initial study population. Moreover, the limited sample size of our study may limit the generalizability of our findings. Therefore, the results should be considered as hypothesis-generating and need confirmation in future studies.”(lines 489-493)

#3 Could the dietary interventions with AAF and AAF-syn have influenced the observed resolution of CMA? It is unclear whether the cytokine changes represent responses to the intervention or natural resolution.

It is unlikely that the dietary interventions with AAF and AAF-syn have influenced the observed resolution of CMA, as no difference in outgrowth of CMA between AAF and AAF-syn was observed in the parent study on PRESTO. We added the following paragraph to the discussion: “No difference in outgrowth of CMA between AAF and AAF-syn was observed in the parent study on PRESTO, and for both AAF and AAF-syn groups tolerance development was in line with natural outgrowth[11]. We observed some differences between AAF and AAF-syn at 6 months (Table 5). The DBPCFC to determine outgrowth of CMA was performed at 12 months. These findings suggest that there may have been differences due to the treatment, although they do not provide evidence of a relationship with improved outgrowth of CMA.”(lines 481-486)

#4 The study mainly analyzes individual cytokine trajectories but does not include between-group comparisons or integrative analyses. Is there a reason why integrative or multivariate approaches were not applied to compare cytokine profiles across groups?

Thank you for suggesting to include also a multivariate analysis. In our revised manuscript we also included a multivariate analysis with Repeated Measures ANOVA–Simultaneous Component Analysis Plus (RM-ASCA+). The method is described in the subsection “Statistical analysis” of the Methods section (lines 164-169). The results are reported in the sections “Development of immune factors over visits within allergy groups” (lines 183-207) and “Development of immune factors over visits within treatment groups” in the Results section.(lines 239-267)
